# Effects of a lipid-based nutrient supplement during pregnancy and lactation on maternal plasma fatty acid status and lipid profile: Results of two randomized controlled trials^☆^

**DOI:** 10.1016/j.plefa.2017.01.007

**Published:** 2017-02

**Authors:** Brietta M. Oaks, Rebecca R. Young, Seth Adu-Afarwuah, Ulla Ashorn, Kristina H. Jackson, Anna Lartey, Kenneth Maleta, Harriet Okronipa, John Sadalaki, Lacey M. Baldiviez, Setti Shahab-Ferdows, Per Ashorn, Kathryn G. Dewey

**Affiliations:** a Program in International and Community Nutrition, Department of Nutrition, University of California, 3135 Meyer Hall, One Shields Avenue, Davis, CA 95616, USA; b Department of Nutrition and Food Science, University of Ghana, Legon, Ghana; c Center for Child Health Research, University of Tampere School of Medicine and Tampere University Hospital, Tampere, Finland; d OmegaQuant Analytics, LLC, Sioux Falls, SD, USA; e University of Malawi College of Medicine, Department of Community Health, Blantyre, Malawi; f USDA, ARS Western Human Nutrition Research Center, Davis, CA, USA; g Department of Paediatrics, Tampere University Hospital, Tampere, Finland

**Keywords:** ALA, α-linolenic acid, DHA, docosahexaenoic acid, DPA, docosapentaenoic acid, EPA, eicosapentaenoic acid, GC, gas chromatography, GLA, γ -linolenic acid, LA, linoleic acid, n-6, omega-6, n-3, omega-3, PUFA, polyunsaturated fatty acids, Pregnancy, Lactation, Omega-3 fatty acids, Supplementation, Lipids, Cholesterol

## Abstract

It is unknown whether a novel small-quantity lipid-based nutrient supplement (SQ-LNS) containing alpha-linolenic (ALA) and linoleic acids impacts maternal plasma lipids and fatty acid status. We measured plasma fatty acids (wt%) and lipid concentrations at 36 wk gestation and breast milk fatty acids (wt%) at 6 months postpartum in a subsample of women enrolled in a randomized controlled trial studying the effects of SQ-LNS on birth outcomes and child growth. Women≤20 wk gestation in Ghana (n=1,320) and Malawi (n=1,391) were assigned to receive daily either: 1) iron-folic acid (pregnancy); 2) multiple micronutrients (pregnancy and lactation); or 3) SQ-LNS (pregnancy and lactation). At 36 wk, plasma ALA levels were higher in those receiving SQ-LNS. SQ-LNS increased breast milk ALA in Ghana but not Malawi. There was no effect on plasma lipids or other selected fatty acids. SQ-LNS may impact plasma and breast milk ALA levels depending on the population.

## 1 Introduction

Adequate amounts of the essential polyunsaturated fatty acids (PUFAs) alpha-linolenic acid (ALA, omega-3) and linoleic acid (LA, omega-6) are required during pregnancy and lactation for optimal fetal and infant growth [1]. Fatty acids are transferred to the fetus during pregnancy through the placenta and continue to be provided during infancy through the mother's milk. Thus, fatty acid consumption and body stores of the mother have a direct effect on fetal and infant fatty acid status [1]. Fatty acid supplementation trials in pregnant populations have primarily focused on the long-chain fatty acid derivatives of ALA – docosahexaenoic acid (DHA) and eicosapentaenoic acid (EPA) [2] – due to the accumulation of DHA in the brain and retina and the low conversion rate of ALA to DHA (approximately 9% of ALA converts to DHA in women) [3]. However, several studies in non-pregnant populations suggest that biomarkers of ALA status are associated with health benefits independent of DHA [4], [5].

Only one trial to date, conducted in the Netherlands, has examined the effect of maternal ALA supplementation on fatty acid status during pregnancy [6]. ALA supplementation led to higher concentrations of ALA, eicosapentaenoic acid (EPA), and docosapentaenoic acid (DPA) (wt%) in maternal plasma at delivery but had no effect on DHA or AA. A summary of ALA supplementation studies (all non-pregnant participants, although some studies included breastfeeding women) also indicated that ALA supplementation generally resulted in increases in plasma and breast milk ALA but had little effect on DHA [7]. However, the majority of these studies have been conducted in Europe and North America, and it is possible that populations with poorer nutritional status might respond differently to ALA supplementation, as various factors can affect the conversion of ALA to DHA (e.g., diets in Europe and North America are high in LA, which inhibits conversion of ALA to DHA) [8]. In many populations, total energy intake among pregnant and lactating women may be adequate, but the ALA and AA content of the usual diet may be low [9]. While estimates specific to ALA and AA are not available, it is estimated that the availability of omega-3 fatty acids in the food supply of Ghana (<0.4% of energy supply) and Malawi (<0.3% of energy supply) is below the minimum recommended level (0.5% of energy supply) to meet the needs of pregnant and lactating women in the populations [9].

Additionally, low maternal plasma cholesterol during pregnancy has been associated with adverse birth outcomes, such as preterm birth [10], [11]. Cholesterol is required for the structural integrity of cell membranes and as a precursor to steroid hormones [12], [13], and is therefore essential during pregnancy for fetal cell formation and placental steroid hormone production [14]. At the same time, high cholesterol and triglycerides are also associated with adverse birth outcomes [15], [16]. In Ghana, both low and high cholesterol may be a concern. A trial conducted in Ghana in non-pregnant healthy adults showed that peanut consumption over the course of 30 wk reduced total plasma cholesterol and triglyceride concentrations [17].

Small-quantity lipid-based nutrient supplements (SQ-LNS) are a novel form of nutritional supplementation that deliver micronutrients in a food-base along with protein, fat, and essential fatty acids (EFA). SQ-LNS are currently being studied as a home fortificant in lower- and middle-income countries to prevent malnutrition, particularly in women and children. We reported previously that compared with iron-folic acid (IFA) and multiple micronutrient (MMN) capsules, providing a peanut-based SQ-LNS to pregnant and lactating women in Ghana promoted fetal growth in vulnerable women, such as primiparas, and had a positive effect on child growth [18], [19], but had no significant effect on newborn birth size or child growth in Malawi [20], [21]. While several other studies have also examined the impact of SQ-LNS on birth size or child growth [22], [23], [24], no study has yet examined whether SQ-LNS have an effect on biomarkers of maternal fatty acid status, even though a key aspect that distinguishes SQ-LNS from other nutritional supplements proposed for lower- and middle-income countries is that it contains EFA.

The objective of the present study was to determine the effects of SQ-LNS provided to women during pregnancy and lactation on maternal plasma fatty acids and lipids and breast milk fatty acids. To address this objective, we compared plasma fatty acids and lipid concentrations at 36 wk gestation and breast milk fatty acids at 6 mo postpartum of women receiving SQ-LNS during pregnancy and lactation with those of women receiving either IFA (during pregnancy only) or MMN (during pregnancy and lactation). Our primary hypotheses were that compared to women who received IFA or MMN, women who received SQ-LNS during pregnancy and lactation would have: 1) lower mean plasma total cholesterol and triglyceride concentrations and a lower prevalence of low total cholesterol (<10th percentile of IFA group) at 36 wk gestation; and 2) higher levels of ALA and LA in plasma at 36 wk gestation and in breast milk at 6 mo postpartum.

## 2 Participants and methods

### 2.1 Participants and study design

This was a sub-study of participants from two randomized controlled trials conducted in Malawi and Ghana as part of the International Lipid-Based Nutrient Supplements (iLiNS) Project (www.ilins.org). The primary objective of these trials was to determine the effect of SQ-LNS, provided during pregnancy, lactation, and early childhood, on child growth at 18 months of age, as compared with IFA provided during pregnancy or MMN provided to the mother during pregnancy and the first six months postpartum. Details of the study methods have been reported elsewhere [18], [20], [21]. Briefly, the study teams in Ghana and Malawi recruited women attending prenatal care visits at four health facilities in semi-urban areas of the Yilo Krobo and Lower Manya Krobo districts about 70 km north of Accra, Ghana between December 2009 and December 2011, and four health facilities in the rural Mangochi district in southern Malawi between February 2011 and August 2012. While the trials were similar in design, each trial operated independently and there were some differences in inclusion/exclusion criteria. In Ghana, women were eligible if they were: ≤20 wk gestation (confirmed by ultrasound), ≥18 y of age, had a completed antenatal health card, and signed or thumb-printed informed consent. We excluded women if they were: HIV positive, had asthma, epilepsy, tuberculosis, a chronic disease that required medical attention, did not reside in the defined catchment area, had a milk or peanut allergy, or were participating in another clinical trial. The Malawi trial had similar exclusion and inclusion criteria, however women in Malawi were eligible if they were ≥15 y of age and were not excluded if they were HIV positive. A daily iron and folic acid capsule is the standard of care during pregnancy in both countries. In Malawi, nutritional supplementation before or after pregnancy is not common. In Ghana, women continue to receive the iron and folic acid capsule 6 wk after delivery and receive high dose vitamin A within 8 wk after delivery, and the use of dietary and herbal supplements in the study setting is common. Women were enrolled in the trials at a mean of 16–17 wk gestation and received supplementation through pregnancy and until 6 months postpartum, which represents an average of 56 wk of supplementation.

In both trials, pregnant women were randomized to receive one of the following three daily treatments: 1) iron and folic acid (IFA, a capsule consisting of 60 mg iron and 400 µg folic acid, received during pregnancy only); 2) multiple micronutrients (MMN, a capsule consisting of 18 vitamins and minerals [including 20 mg iron], received during pregnancy and the first 6 months postpartum); or 3) SQ-LNS (a 20 g sachet which included the same 18 micronutrients as the MMN capsule plus four additional minerals: calcium, phosphorus, potassium, magnesium) (see Table 1 for nutrient content of each supplement). Both the IFA and MMN groups were considered control groups. Group allocations were determined by a statistician who used a computer-generated randomization scheme in blocks of 9 (3 codes for each of the 3 interventions) and codes were placed in sealed opaque envelopes. A woman chose an envelope from a stack of envelopes (6 and 9 envelopes per stack in Malawi and Ghana, respectively) to determine her group allocation and received her first 2-wk supplement ration at this time. Field workers made home visits biweekly, during which they delivered the supplements and collected information on the participant's adherence to the study intervention. Adherence was assessed by maternal report as well as by counting the numbers of unconsumed capsules or sachets. This was a partially-blinded trial, as it was not possible to blind the fieldworkers and study participants to those consuming capsules vs. SQ-LNS (because of the starkly different characteristics).

In Ghana, from the 1,320 women enrolled in the trial, 510 were excluded from the present analysis due to an error in the labeling of the IFA and MMN supplements, resulting in mixed exposure [18]. From the remaining 810 women, enrolled from October 2010 to December 2011, 369 women were randomly selected for analysis of blood lipids and fatty acids (Supplementary Fig. 1). In Malawi, from the 1,391 women enrolled in the trial, 315 women were randomly selected for analysis of fatty acids and all 1,391 for blood lipids (Supplementary Fig. 2).

The institutional review boards at the College of Medicine Research, University of Malawi and the Ethics Committee of Pirkanmaa Hospital District, Finland approved the study protocol for the trial in Malawi. The institutional review boards at the University of California, Davis; the Noguchi Memorial Institute for Medical Research, University of Ghana; and the Ghana Health Service approved the study protocol for the trial in Ghana. Both trials were registered at www.clinicaltrials.gov (IDs: NCT01239693, NCT00970866).

### 2.2 Breast milk and blood sample collection

At both sites, study nurses collected venous blood samples from women at enrollment and 36 wk gestation into a heparin-treated, trace element-free, Sarstedt Monovette tube. Because of the difficulty of obtaining fasting blood samples from pregnant women, non-fasting blood samples were collected. However, we collected information regarding time between last meal and sample collection. Lab technicians centrifuged the blood samples at 4000 rpm for 15 min to obtain plasma. Breast milk was collected at 6 months postpartum. In Ghana, study nurses assisted women in collecting a 10–20 mL mid-stream breast milk sample during a follow-up clinic visit. In Malawi, women expressed a full milk sample from a single breast during a home visit. A trained field worker then mixed the breast milk and collected a 10 mL sample, with the remaining milk provided to the infant by spoon. All samples were stored at −20 °C within 24 h of collection and moved to −80 °C for longer term storage.

### 2.3 Cholesterol and triglyceride analysis

Plasma samples from the Ghana trial were analyzed in Accra, Ghana for total cholesterol, HDL-C, and triglyceride concentrations using a Flexor Junior Chemistry Analyzer (Vital Scientific, Dieren, Netherlands). LDL-C was calculated using the Friedewald equation: LDL-C=total cholesterol–(HDL-C)–(triglycerides/5), mg/dL [25]. This is accepted as an accurate method of determining LDL-C concentration, as long as triglyceride concentration is not ≥400 mg/dL [25], [26]; all of our samples were below this level. Plasma samples from the Malawi trial were shipped on dry ice to Davis, CA, where lab technicians quantified total cholesterol and triglyceride concentrations by enzymatic colorimetric assay using a Cobas Integra 400 plus automatic analyzer (Roche Diagnostic Corp., Indianapolis, IN). All lab technicians were blinded to the intervention groups.

### 2.4 Fatty acids analysis

Plasma and breast milk samples from both trials were shipped to OmegaQuant Analytics (Sioux Falls, SD) for analysis of fatty acids. Fatty acid composition was analyzed by gas chromatography with flame ionization detection. Plasma or breast milk was added to a mixture of solvents (methanol containing 14% boron trifluoride: toluene: methanol; 35:30:35 v/v/v, all from Sigma-Aldrich, St. Louis, MO). The tube was vortexed and heated in a hot bath at 100 °C for 45 min. After cooling, hexane (EMD Chemicals, USA) and distilled water were added. The sample was vortexed and centrifuged, and then an aliquot of the hexane phase was analyzed by gas chromatography using a GC-2010 (Shimadzu Corporation, Columbia, MD) equipped with a SP-2560, 100-m fused silica capillary column (0.25 mm internal diameter, 0.2 µm film thickness; Supelco, Bellefonte, PA). Fatty acid composition was expressed as a percent by weight (wt%) of total identified fatty acids. All lab technicians were blinded to the intervention groups.

### 2.5 Maternal characteristics

At enrollment, anthropometrists measured weight and height using high-quality scales and stadiometers. We calculated body mass index (BMI = kg/m^2^). Lab technicians assessed malaria and HIV infection status with rapid tests. Concentrations of inflammatory biomarkers C-reactive protein (CRP, mg/L) and alpha-1 glycoprotein (AGP, g/L) were measured in plasma by immunoturbidimetric assay using the autoanalyzers specified earlier. Trained interviewers collected socioeconomic and demographic information at a follow-up home visit.

### 2.6 Sample size and statistical analysis

For analysis of fatty acids and lipid concentrations, we used a minimum effect size (Cohen's *d*) of 0.5 to calculate sample size, assuming a two-sided α=0.05% and 80% power, requiring a subsample of 79 per group (total n=237). Allowing for attrition and women missing a sample at either baseline or 36 wk gestation, 369 women were randomly selected from the 810 women in Ghana enrolled after October 1, 2010. In Malawi, 315 women were randomly selected from women with blood samples at both baseline and 36 wk gestation and a breast milk sample at 6 months postpartum. The effect of the intervention on cholesterol and triglyceride concentrations was examined in the full sample in Malawi and in the subsample in Ghana.

Statistical analysis was performed according to intention-to-treat and focused on examining differences between the three intervention groups in plasma fatty acid levels and lipid concentrations at 36 wk gestation and breast milk fatty acid levels at 6 months postpartum. The primary fatty acids of interest were ALA and LA. Secondarily, to determine if there was any impact on LCPUFAs, we also examined the effect of supplementation on DHA, EPA, AA, the sum of DHA and EPA, the sum of all long chain omega-3 fatty acids (DHA, EPA, and DPA), and the following ratios: LA:AA, ALA:DHA, AA:EPA, and omega-6 fatty acids:omega-3 fatty acids. We limited our analyses to these fatty acids and ratios as indicators of LCPUFA metabolism and due to their potential effects on infant growth and neurodevelopment [1], [3]. Cholesterol and triglycerides were analyzed as concentrations, and fatty acids were analyzed as percentage of total fatty acids (by weight). Logarithmic transformation of all fatty acid variables and triglyceride concentration was applied to approximate a normal distribution of the data which was evaluated using the Shapiro-Wilk test. Fatty acids were dichotomized into high or low values using a median cut-point and low cholesterol was defined as <10th percentile of the IFA group at 36 wk gestation. We also used the following clinical definitions: high total cholesterol (≥240 mg/dL), high LDL-C (≥160 mg/dL), and low HDL-C (<50 mg/dL) [27], [28].

We used the Household Food Insecurity Access Scale [29] to estimate food insecurity and created scores using standard criteria adjusted for the month of collection. An asset index was created using principal components analysis [30] based on household ownership of a set of assets (radio, television, cell phone, bed, mattress, bed net, and bicycle), lighting source, drinking water supply, sanitation facilities, and flooring materials.

We evaluated the effect of the nutritional intervention on lipids and fatty acids in plasma using ANCOVA (continuous outcomes) and logistic regression (binary outcomes) models, using the Tukey-Kramer adjustment for multiple comparisons and p<0.05 indicating statistical significance. We performed analyses both with and without covariates, as guidelines for best statistical practices support the use of covariates in analyses of randomized controlled trials [31]. All models included the baseline value for the outcome variable. This is mathematically the same as testing for a difference in the change in the outcome (between baseline and 36 wk) between the three groups. We used similar statistical analyses for fatty acids in breast milk, although those models could not include a baseline value, and we only had one time point for breast milk sample collection. Pooled analyses included trial site in all models. We considered covariates for inclusion in the model if they were associated with the outcome variable at p<0.1. Potential covariates were selected from previous literature and stated in a predefined analysis plan; they included baseline measurement of the outcome variable, gestational age, maternal age, education level, BMI and height, season of enrollment, malaria infection, HIV status (Malawi only), inflammatory markers, household food insecurity, asset index, parity, site of enrollment, and time since last meal.

To determine whether a pooled data analysis could be conducted, we tested for interaction between study site and intervention group. We also tested for interaction between the intervention group and maternal age, parity, and baseline BMI. All interactions were evaluated in linear regression models and interaction term p-values<0.1 were considered to be statistically significant. We compared baseline characteristics between women in each of the three intervention groups, as well as between those with complete data and those with missing data at 36 wk gestation, as this time point has the largest proportion of missing data. In sensitivity analyses, we performed unadjusted analyses using the same sample that was used in adjusted analyses, and considered a >10% change in results to be indicative of bias. We also performed another sensitivity analysis to compare those receiving LNS with those not receiving LNS (IFA group and MMN group combined) for all outcomes. Model assumptions were checked using standard regression diagnostics for linearity, normality, leverage, and influence. All analyses were performed using SAS 9.4 (SAS Institute, Cary, NC).

## 3 Results

At enrollment, plasma fatty acid data were available for 628 women (313 in Ghana, 315 in Malawi) and plasma lipid data for 1,718 women (347 in Ghana, 1,371 in Malawi). At 36 wk gestation, plasma fatty acid and lipid data from follow-up visits were available for 536 (221 in Ghana, 315 in Malawi) and 1,365 (298 in Ghana, 1,067 in Malawi) women, respectively. At 6 months postpartum, breast milk fatty acid data were available for 618 women (303 in Ghana, 315 in Malawi). Loss-to-follow-up did not differ by intervention group in either trial. We compared women with missing data at 36 wk gestation with those who had complete data (Supplementary Table 1**)**. Women with complete blood lipid data in Malawi differed in some baseline characteristics and had lower mean baseline cholesterol (121 vs. 125 mg/dL, p=0.04) and triglyceride (95 vs. 101 mg/dL, p=0.02) concentrations than those with complete data. Women in Ghana with complete plasma fatty acid data had higher mean concentration of DHA at baseline (5.1 vs. 4.9%, p=0.04), but were otherwise similar.

**Fig. 1:**
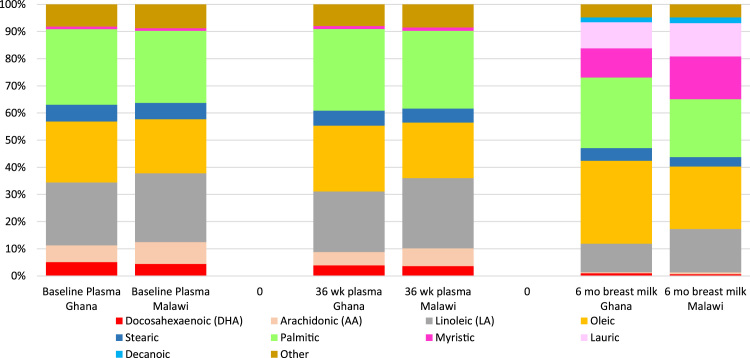
Fatty acid profile of maternal plasma at enrollment and 36 wk gestation and breast milk at 6 mo postpartum.

**Fig. 2:**
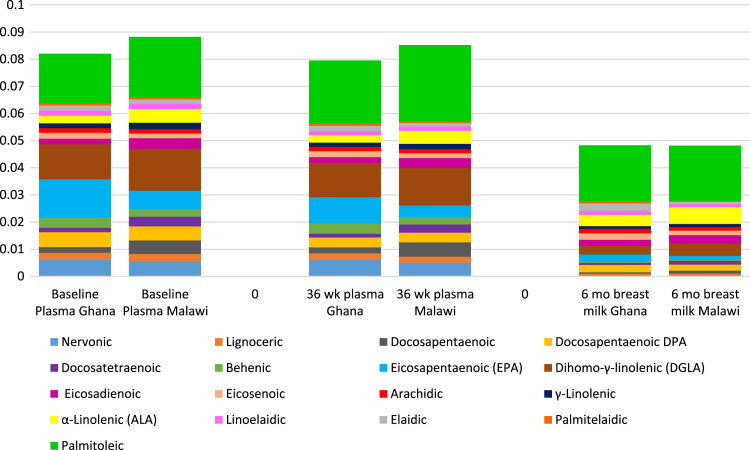
‘Other’ fatty acids category from Fig. 1 expanded.

Baseline maternal characteristics did not differ by intervention group in either site (Table 2**)**. Baseline values for blood lipids and fatty acids were generally similar among intervention groups within each trial, although AA and triglyceride levels were higher in the IFA group in Malawi (Table 3). There was no significant interaction between study site and intervention group, which allowed for pooled analyses. However, given the differences in baseline fatty acid and blood lipid levels between the two trials, analyses were also conducted for each trial separately. A comparison of the fatty acid profiles of women in the two countries is presented in Fig. 1 and Fig. 2.

At 36 wk gestation in the Malawi trial, a group difference in mean plasma ALA was evident in the adjusted model (p=0.04), with women receiving SQ-LNS demonstrating higher values compared with women receiving MMN (SQ-LNS: 0.49, MMN: 0.44 wt%, Tukey-adjusted p=0.04). In the Ghana trial, a significant group difference was seen in the ALA:DHA ratio in plasma at 36 wk gestation (p=0.03) and breast milk at 6 months postpartum (p=0.03) (fatty acid ratio results in Supplementary Table 2**)**, with significant differences of the ALA:DHA ratio between SQ-LNS and IFA in plasma (ratio: 0.07 vs. 0.06, p=0.03) and between SQ-LNS and MMN in breast milk (ratio: 0.48 vs. 0.39, p=0.02). There was a significant difference in mean plasma ALA in pooled analyses (p=0.04) (Table 3), which remained significant in analyses adjusted for selected covariates (p=0.02). The SQ-LNS group had higher ALA levels compared with either the group receiving MMN (SQ-LNS: 0.38, MMN: 0.35% wt, Tukey-adjusted p=0.06) or IFA (SQ-LNS: 0.38, IFA: 0.36% wt, Tukey-adjusted p=0.08). No other differences in fatty acid levels or ratios or blood lipid concentrations between groups were significant. There was no difference in the proportion of women with low plasma cholesterol (<10th percentile of IFA group) across the intervention groups in either unadjusted or adjusted analyses.

At 6 months postpartum, women receiving SQ-LNS in the Ghana trial had higher ALA levels in breast milk (p=0.02) and a higher ratio of ALA:AA (SQ-LNS: 1.07, MMN: 0.95: Tukey-adjusted p=0.02) in breast milk compared with women receiving MMN. See Table 4 for selected results. These differences became non-significant in adjusted analyses (data not shown). In the Malawi trial and pooled analyses, breast milk fatty acid levels and fatty acid ratios were not significantly different across intervention groups in either unadjusted or adjusted analyses.

## 4 Discussion and conclusion

### 4.1 Discussion

As SQ-LNS are novel nutritional supplements, this research aimed to determine the effect of SQ-LNS during pregnancy and lactation on maternal plasma fatty acid status and lipid concentrations. We found no significant difference in plasma fatty acid levels or lipid concentrations in Ghana or Malawi at 36 wk gestation, although pooled results suggest that SQ-LNS supplementation increases mean maternal plasma ALA levels during pregnancy. SQ-LNS increased ALA levels in breast milk in Ghana but did not impact breast milk fatty acid levels in Malawi.

In terms of DHA, our results are consistent with previous ALA supplementation trials. A review by Brenna et al. reported that out of 21 such trials, only 7 trials reported an effect on DHA [7], although other omega-3 LCPUFAs (EPA, DPA) were typically higher in plasma from participants receiving ALA. It is possible that the lack of an effect on LCPUFAs is due to the amount of ALA (0.59 g) in SQ-LNS, as all of the trials in the review used higher ALA doses (range: 1.5–40 g). It is also possible that SQ-LNS had a limited impact on plasma and breast milk fatty acids because it also included 4.59 g LA. A recent review of the effect of dietary modification of ALA and LA showed that reducing LA intake was a key factor for increasing omega-3 LCPUFA levels [32].

It is likely that women in the study area in Malawi regularly consume fish high in DHA and AA and plant oils that are high in LA and low in ALA [33]. Food and Agriculture Organization (FAO) balance sheets also support the possibility of high fish consumption in Ghana and use of high LA/low ALA oils [9]. Breast milk DHA levels were 2 and 3 times higher in Malawi and Ghana, respectively, than the worldwide average (0.32±0.22% [full range: 0.06–1.40%]) established by Brenna et al. [34] As a point of reference, Ghanaian milk DHA levels were comparable to Japanese levels, where fish intake is very high. Both African cohorts had substantially higher milk DHA levels than in the US (~0.2% [35]) and other industrialized countries. The milk AA values were within the normal range described by Brenna et al. [34] (0.47±0.13% [full range: 0.24–1.0%]), with the Malawi values on the higher end and the Ghana values on the lower end of normal. It is possible that SQ-LNS had an impact on breast milk ALA in Ghana but no other fatty acids due to low dietary intake of ALA but high dietary intake of DHA. It is unclear why there was not a similar effect in Malawi. Women in the Malawi trial had higher breast milk ALA than those in Ghana, perhaps indicating less ability to respond to additional ALA. The women in the Malawi trial also had poorer indicators of health status (higher prevalence of anemia and malaria), which may have resulted in reduced correlation between fatty acid intake and fatty acid biomarkers in plasma. A recent study showed that in a Swedish population in which seafood intake was correlated with serum DHA, there was no such correlation among individuals with atopic eczema [36], raising the possibility that other health conditions might be associated with discordance between fatty acid intake and biomarkers.

The different levels of micronutrients provided in the three different supplements may have also impacted women's fatty acid status (i.e., excess iron can lead to lipid oxidation of fatty acids [37]; antioxidants, such as vitamin E [α-tocopherol], can prevent oxidation of fatty acids [38]). MMN and SQ-LNS had the same micronutrient content, except that SQ-LNS also contained magnesium, phosphorus, calcium, and potassium. While it is unlikely the addition of phosphorus, calcium, or potassium affected fatty acid levels, some research in animal models has demonstrated that deficiency in magnesium can result in lower PUFA concentrations [39].

Peanut consumption has been associated with improved plasma lipid profiles in several studies, including a trial in Ghana [17], [40], [41]. Given that SQ-LNS is peanut-based, we had hypothesized that SQ-LNS may impact plasma lipid profiles of the women receiving this supplement. There are several possible explanations for the lack of an effect of SQ-LNS on plasma lipids in this study. First, it is possible that the dose of SQ-LNS was too small to produce an effect. Second, the majority of the plasma lipid concentrations at enrollment were within the normal range for healthy adults, so it is possible that there was limited opportunity for improvement. Lastly, cholesterol and triglyceride concentrations increase over the course of pregnancy [14] and therefore the cholesterol and triglyceride lowering-effect associated with peanut consumption might be masked by the increase in both of these plasma lipids during pregnancy.

Strengths of this study include rigorous follow-up and monitoring of compliance, the large sample size within a randomized controlled trial study design, and the long duration of supplementation. Women were enrolled in the trials at a mean of 16–17 wk gestation and received supplementation through pregnancy and up to 6 months postpartum, which approximates 56 wk of supplementation. Previously, the longest duration of ALA supplementation in a trial that measured fatty acid status was 42 wk [7], [42]. Duration of ALA supplementation has been shown to be a factor with regard to effects on LCPUFAs, as a trial in Japanese men reported no effect in DHA at 3 mo, but increased levels of DHA after 10 mo of supplementation [42].

There are limitations to this study. Our trials experienced loss to follow-up, however we conducted sensitivity analyses and did not find evidence of this leading to a bias in results, except for a bias towards the null for breast milk fatty acids from the Ghana trial. It was not possible to blind women to whether they were assigned SQ-LNS vs. a capsule, however we think it is unlikely that this partial blinding influenced these results as lab technicians were blinded. Different laboratories were used to analyze the plasma lipids and different methods of breast milk collection were used between the two trials, so we cannot exclude the possibility that differences in plasma lipids or breast milk fatty acid levels between the two populations may be due to method of laboratory analysis and collection, respectively. However, expressing fatty acid levels as the percentage of total fatty acids by weight should eliminate bias due to differences in total milk fat concentrations. Fatty acids were analyzed in plasma as opposed to erythrocytes, which may have led to greater within-person variability [43]. Plasma fatty acids are more strongly influenced by recent intake compared to erythrocytes [44]. Because of the difficulty of obtaining fasting blood samples from pregnant women, non-fasting blood samples were collected. However, we collected information regarding time between last meal and sample collection and included this in adjusted models when significant (p<0.1). Typically, there is minimal effect of fasting on total cholesterol, HDL-C, and LDL-C, although triglycerides tend to be approximately 15% higher in non-fasting vs. fasting samples [45], [46]. Lastly, we examined multiple outcomes and thus our results demonstrating an impact of SQ-LNS on plasma and breast milk ALA levels may be due to chance. However, we note that the supplement contained ALA and previous studies support the biological plausibility of an effect on ALA levels without an effect on other fatty acid levels.

### 4.2 Conclusion

The current formulation of SQ-LNS may increase plasma ALA in late pregnancy in settings similar to the study sites in Malawi and Ghana, and may impact breast milk ALA levels in populations similar to the study population in Ghana. Research to evaluate different formulations of SQ-LNS, particularly with higher amounts of ALA and lower amounts of LA, would be beneficial for further examining the potential of SQ-LNS to affect maternal fatty acid status.

## Conflicts of interest

KHJ is employed by OmegaQuant Analytics, LLC, a commercial laboratory that performed the fatty acid analysis for this study. The other authors declare no conflicts of interest related to this study.

## Names for PubMed indexing

Oaks, Dewey, Young, Adu-Afarwuah, Ashorn, Jackson, Lartey, Maleta, Okronipa, Sadalaki, Baldiviez, Shahab-Ferdows, Vosti, Ashorn

## Trial registration

ClinicalTrials.gov, Identifiers NCT00970866 (Ghana trial) and NCT01239693 (Malawi trial).

## Figures and Tables

**Table 1:** Nutrient composition of supplements used in the study: iron and folic acid (IFA) capsule, multiple micronutrient (MMN) capsule, and small-quantity lipid-based nutrient supplement (SQ-LNS).

**Nutrient**	**IFA**	**MMN**	**SQ-LNS (20 g)**
Energy (kcal)	0	0	118
Protein (g)	0	0	2.6
Fat (g)	0	0	10
Linoleic acid (g)	0	0	4.59
α-linolenic acid (g)	0	0	0.59
Iron (mg, ferrous sulphate)	60	20	20
Folic acid (µg, pteroyl monoglutamic acid)	400	400	400
Vitamin A (µg RE, retinyl acetate)	0	800	800
Vitamin B12 (µg, cyanocobalamin 0.1%)	0	5.2	5.2
Vitamin B6 (mg, pyridoxine hydrochloride)	0	3.8	3.8
Vitamin C (mg, L-ascorbic acid)	0	100	100
Vitamin D (IU, cholecalciferol [D3])	0	400	400
Zinc (mg, zinc sulphate)	0	30	30
Thiamin (mg, thiamin hydrochloride)	0	2.8	2.8
Riboflavin (mg, riboflavin)	0	2.8	2.8
Niacin (mg, niacinamide)	0	36	36
Vitamin E (mg, DL-α-tocopherol acetate)	0	20	20
Vitamin K (µg, phylloquinone 5%)	0	45	45
Pantothenic acid (mg, calcium pantothenate)	0	7	7
Copper (mg, encapsulated copper sulphate)	0	4	4
Iodine (µg, potassium iodate)	0	250	250
Manganese (mg, manganese sulphate)	0	2.6	2.6
Selenium (µg, sodium selenite 1.5%)	0	130	130
Calcium (mg, tricalcium phosphate)	0	0	280
Phosphorus (mg, tricalcium phosphate)	0	0	190
Potassium (mg, potassium chloride)	0	0	200
Magnesium (mg, magnesium citrate)	0	0	65
Phytate (mg)	0	0	24.7

**Table 2:** Baseline characteristics of the participating women at enrollment by study group.^a^

	**Ghana**				**Malawi**			
**Characteristic**	**IFA (n=124)**	**MMN (n=121)**	**SQ-LNS (n=124)**	**P**^c^	**IFA (n=457)**	**MMN (n=463)**	**SQ-LNS (n=453)**	**P**^c^
Maternal age, years	26.3 (5.0)	26.3 (5.5)	27.0 (5.4)	0.53	25.0 (6.1)	24.8 (6.1)	25.1 (6.2)	0.79
Education, completed years	7.4 (3.3)	7.2 (3.3)	7.9 (3.7)	0.19	3.9 (3.4)	4.1 (3.4)	4.1 (3.6)	0.57
Primiparous women	38.9%	34.9%	31.6%	0.46	20.4%	23.2%	22.4%	0.58
Gestational age at enrollment, weeks	16.3 (3.3)	16.2 (3.0)	16.2 (3.1)	0.97	16.8 (2.1)	16.8 (2.1)	16.9 (2.2)	0.91
Body mass index (BMI), kg/m^2^	24.7 (4.2)	24.7 (4.1)	24.8 (3.8)	0.98	22.1 (2.6)	22.2 (2.9)	22.2 (3.0)	0.58
Women with anemia (Hb<100 g/l)	11.5%	16.7%	13.5%	0.47	21.2%	20.0%	21.4%	0.84
Women overweight or obese BMI (≥25 kg/m^2^)	42.3%	40.3%	39.2%	0.88	13.2%	12.4%	13.3%	0.91
Women with a low BMI (<18.5 kg/m^2^)	2.9%	1.6%	0.8%	0.46	6.0%	4.6%	5.8%	0.60
Women with a positive HIV test^b^	–	–	–	–	15.5%	11.2%	14.2%	0.15
Women with a positive malaria test (RDT)	6.5%	4.8%	9.0%	0.42	22.3%	24.2%	23.3%	0.79

a Mean (SD) except where noted.

b HIV positive women were excluded from the trial in Ghana.

c p-value obtained from ANOVA (comparison of means) or Fisher's exact test (comparison of proportions).

**Table 3:** Median plasma ALA, LA, DHA, AA, and n6:n3 ratio and mean maternal plasma lipids by intervention group.

	**Ghana**				**Malawi**				**Pooled**
	**IFA**	**MMN**	**SQ-LNS**	**p**^g^	**IFA**	**MMN**	**SQ-LNS**	**p**^g^	**p**^g^
**Fatty Acids**	**(n=110)**^c^	**(n=98)**^c^	**(n=105)**^c^		**(n=103)**	**(n=106)**	**(n=106)**		
**ALA, wt%**									
Enrollment	0.28	0.26	0.25	0.07	0.46	0.50	0.42	0.22	0.21
	(0.23, 0.34)^d^	(0.23, 0.31)	(0.22, 0.32)		(0.35, 0.61)	(0.38, 0.64)	(0.37, 0.54)		
36 wk^a^	0.26	0.26	0.27	0.10	0.47	0.45	0.48	0.18	0.04^h^
	(0.22, 0.30)	(0.21, 0.32)	(0.23, 0.32)		(0.35, 0.59)	(0.35, 0.55)	(0.37, 0.59)		
**LA, wt%**									
Enrollment	23.9	23.5	23.1	0.27	25.4	25.6	25.6	0.42	0.18
	(21.6, 25.0)	(21.6, 25.0)	(21.3, 24.6)		(22.9, 27.1)	(23.3, 28.1)	(22.6, 27.7)		
36 wk^a^	22.0	22.1	22.6	0.13	25.2	25.8	25.6	0.28	0.14
	(20.1, 23.7)	(20.2, 24.1)	(20.5, 24.6)		(23.3, 27.5)	(23.7, 28.5)	(23.1, 28.5)		
**DHA, wt%**									
Enrollment	5.08	4.99	5.06	0.29	4.62	4.28	4.38	0.62	0.34
	(4.39, 5.65)	(4.41, 5.63)	(4.61, 5.81)		(3.65, 5.17)	(3.53, 5.01)	(3.75, 5.00)		
36 wk^a^	4.01	3.72	3.87	0.56	3.59	3.61	3.57	0.95	0.80
	(3.33, 4.64)	(3.27, 4.39)	(3.41, 4.54)		(3.01, 4.13)	(2.89, 4.24)	(3.07, 4.14)		
**AA, wt%**									
Enrollment	6.09	6.24	5.90	0.24	8.30	7.74	7.89	0.04	0.52
	(5.38, 6.87)	(5.69, 7.23)	(5.34, 6.60)		(7.17, 9.36)	(7.03, 8.53)	(7.11, 8.88)		
36 wk^a^	4.94	5.09	5.12	0.37	6.68	6.60	6.41	0.12	0.68
	(4.45, 5.48)	(4.29, 5.69)	(4.51, 5.56)		(5.91, 7.54)	(5.87, 7.21)	(5.66, 7.04)		
**n6:n3**									
Enrollment	4.5	4.5	4.3	0.19	5.9	6.3	6.2	0.56	0.20
	(3.9, 5.3)	(3.8, 5.4)	(3.8, 4.9)		(5.0, 7.5)	(5.0, 7.5)	(5.1, 7.2)		
36 wk^a^	5.4	5.6	5.5	0.35	7.3	7.2	7.3	0.79	0.59
	(4.4, 6.4)	(4.7, 7.2)	(4.6, 6.5)		(6.1, 8.8)	(6.0, 9.1)	(6.0, 8.6)		
					
**Plasma Lipids**	**(n=115)**^e^	**(n=117)**^e^	**(n=115)**^e^		**(n=457)**^e^	**(n=463)**^e^	**(n=453)**^e^		
**Cholesterol, mg/dL**									
Enrollment	144.0±33.3^f^	142.6±32.2	145.2±37.7	0.85	122.1±32.4	121.3±30.3	120.9±30.7	0.84	0.89
36 wk	165.5±41.0	163.5±40.2	166.6±43.5	0.86	149.6±36.1	151.0±39.0	150.9±37.2	0.86	0.87
**HDL-C, mg/dL**^b^									
Enrollment	56.3±21.5	54.5±21.3	59.0±24.2	0.31	–	–	–	–	–
36 wk	66.6±29.7	66.0±29.3	67.7±31.6	0.92	–	–	–	–	–
**LDL-C, mg/dL**^b^									
Enrollment	63.5±28.7	64.3±25.5	63.7±29.0	0.98	–	–	–	–	–
36 wk	68.8±32.9	68.8±31.8	68.8±34.9	1.00	–	–	–	–	–
**Triglycerides, mg/dL**									
Enrollment	125.9±57.3	114.2±55.7	115.4±59.1	0.23	99.8±43.1	94.1±34.6	94.5±35.8	0.04	0.01
36 wk	146.8±76.1	144.2±70.4	149.9±74.9	0.86	150.8±62.6	147.4±61.4	150.0±63.6	0.75	0.68

a Model includes baseline value of outcome variable for reported p-value.

b HDL-C and LDL-C were not measured in Malawi.

c For plasma fatty acids at 36 wk gestation in Ghana, IFA: n=69, MMN: n=77, SQ-LNS: n=75

d Median (25th percentile, 75th percentile), all such values.

e For plasma lipids at 36 wk gestation in Ghana, IFA: n=95, MMN: n=104, SQ-LNS: n=99, and in Malawi, IFA: n=352, MMN: n=362, SQ-LNS: n=352.

f Mean±SD, all such values.

g P-values obtained by ANOVA (baseline analyses) and ANCOVA (36 wk gestation analyses).

h Two pairwise comparisons had a p-value<0.10: 1) SQ-LNS vs. MMN, Tukey-adjusted p=0.06; and 2) SQ-LNS vs. IFA, Tukey-adjusted p=0.08.

**Table 4:** Median breast milk ALA, LA, DHA, AA, and n6:n3 ratio by intervention group at 6 months postpartum.

	**Ghana**				**Malawi**				**Pooled**
	**IFA (n=102)**	**MMN (n=100)**	SQ-**LNS (n=101)**	**p**^a^	**IFA (n=103)**	**MMN (n=106)**	SQ-**LNS (n=106)**	**p**^a^	**p**^a^
**ALA, wt%**	0.41^b^	0.38	0.44	0.02	0.59	0.63	0.64	0.33	0.11
	(0.39, 0.44)	(0.36, 0.40)	(0.40, 0.47)		(0.54, 0.63)	(0.57, 0.68)	(0.59, 0.69)		
**LA, wt%**	10.4	10.3	10.9	0.14	15.7	16.5	15.8	0.32	0.41
	(10.0, 10.7)	(10.0, 10.6)	(10.4, 11.4)		(14.9, 16.5)	(15.7, 17.2)	(15.1, 16.5)		
**DHA, wt%**	0.97	0.95	0.89	0.54	0.60	0.68	0.65	0.49	0.33
	(0.86, 1.07)	(0.87, 1.03)	(0.81, 0.98)		(0.55, 0.65)	(0.61, 0.75)	(0.57, 0.72)		
**AA, wt%**	0.40	0.39	0.39	0.73	0.63	0.62	0.61	0.65	0.48
	(0.38, 0.42)	(0.37, 0.40)	(0.37, 0.41)		(0.60, 0.65)	(0.59, 0.64)	(0.59, 0.64)		
**n6:n3**	7.2	7.0	7.4	0.57	12.4	12.4	11.8	0.50	0.80
	(6.6, 7.8)	(6.5, 7.5)	(6.8, 7.9)		(11.6, 13.2)	(11.3, 13.5)	(11.0, 12.6)		

a P-values obtained by ANOVA.

b Median (25th percentile, 75th percentile), all such values.

## Appendix A Supplementary material

Supplementary material Supplementary Figure 1. Participant flowchart for lipid and fatty acid analyses in the iLiNS-DYAD trial in Ghana.

.

Supplementary material Supplementary Figure 2. Participant flowchart for lipid and fatty acid analyses in iLiNS-DYAD trial in Malawi.
