# Supplementary material for: Effects of a lipid-based nutrient supplement during pregnancy and lactation on maternal plasma fatty acid status and lipid profile: Results of two randomized controlled trials
Source: Prostaglandins Leukot Essent Fatty Acids. 2017 Feb;117:28–35. doi: 10.1016/j.plefa.2017.01.007 (PMC5338685; doi:10.1016/j.plefa.2017.01.007)
Supplement: Supplementary file 1 — Supplementary material Supplementary Figure 1. Participant flowchart for lipid and fatty acid analyses in the iLiNS-DYAD trial in Ghana. [file mmc1.pptx]

## Slide 1
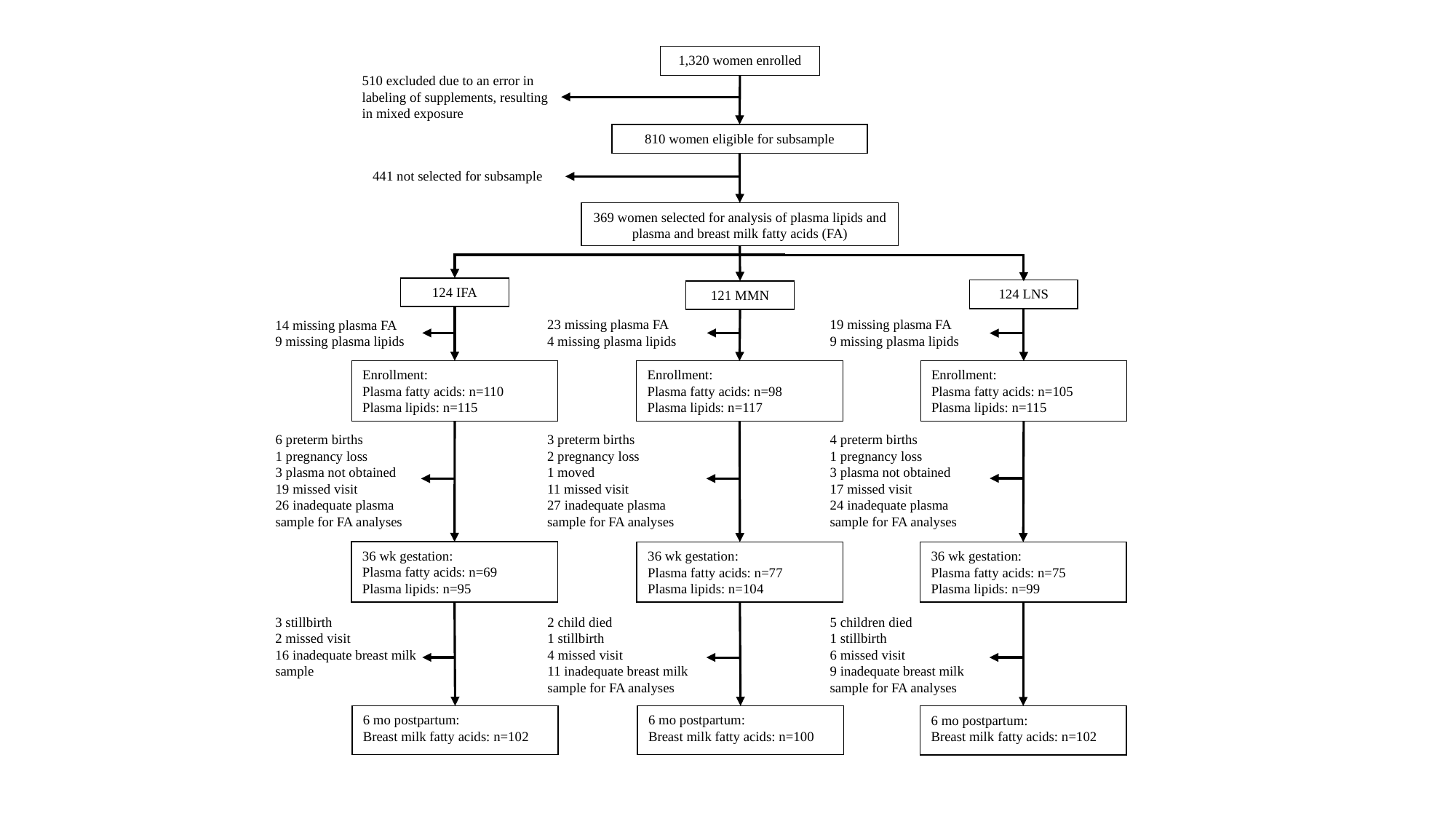

1,320 women enrolled
510 excluded due to an error in labeling of supplements, resulting in mixed exposure
810 women eligible for subsample
441 not selected for subsample
369 women selected for analysis of plasma lipids and plasma and breast milk fatty acids (FA)
124 IFA
124 LNS
121 MMN
14 missing plasma FA
9 missing plasma lipids
23 missing plasma FA
4 missing plasma lipids
19 missing plasma FA
9 missing plasma lipids
Enrollment:
Plasma fatty acids: n=110
Plasma lipids: n=115
Enrollment:
Plasma fatty acids: n=98
Plasma lipids: n=117
Enrollment:
Plasma fatty acids: n=105
Plasma lipids: n=115
6 preterm births
1 pregnancy loss
3 plasma not obtained
19 missed visit
26 inadequate plasma sample for FA analyses
3 preterm births
2 pregnancy loss
1 moved
11 missed visit
27 inadequate plasma sample for FA analyses
4 preterm births
1 pregnancy loss
3 plasma not obtained
17 missed visit
24 inadequate plasma sample for FA analyses
36 wk gestation:
Plasma fatty acids: n=69
Plasma lipids: n=95
36 wk gestation:
Plasma fatty acids: n=77
Plasma lipids: n=104
36 wk gestation:
Plasma fatty acids: n=75
Plasma lipids: n=99
2 child died
1 stillbirth
4 missed visit
11 inadequate breast milk sample for FA analyses
5 children died
1 stillbirth
6 missed visit
9 inadequate breast milk sample for FA analyses
3 stillbirth
2 missed visit
16 inadequate breast milk sample
6 mo postpartum:
Breast milk fatty acids: n=102
6 mo postpartum:
Breast milk fatty acids: n=100
6 mo postpartum:
Breast milk fatty acids: n=102
