# Supplementary material for: Effects of a lipid-based nutrient supplement during pregnancy and lactation on maternal plasma fatty acid status and lipid profile: Results of two randomized controlled trials
Source: Prostaglandins Leukot Essent Fatty Acids. 2017 Feb;117:28–35. doi: 10.1016/j.plefa.2017.01.007 (PMC5338685; doi:10.1016/j.plefa.2017.01.007)
Supplement: Supplementary file 2 — Supplementary material Supplementary Figure 2. Participant flowchart for lipid and fatty acid analyses in iLiNS-DYAD trial in Malawi. [file mmc2.pptx]

## Slide 1
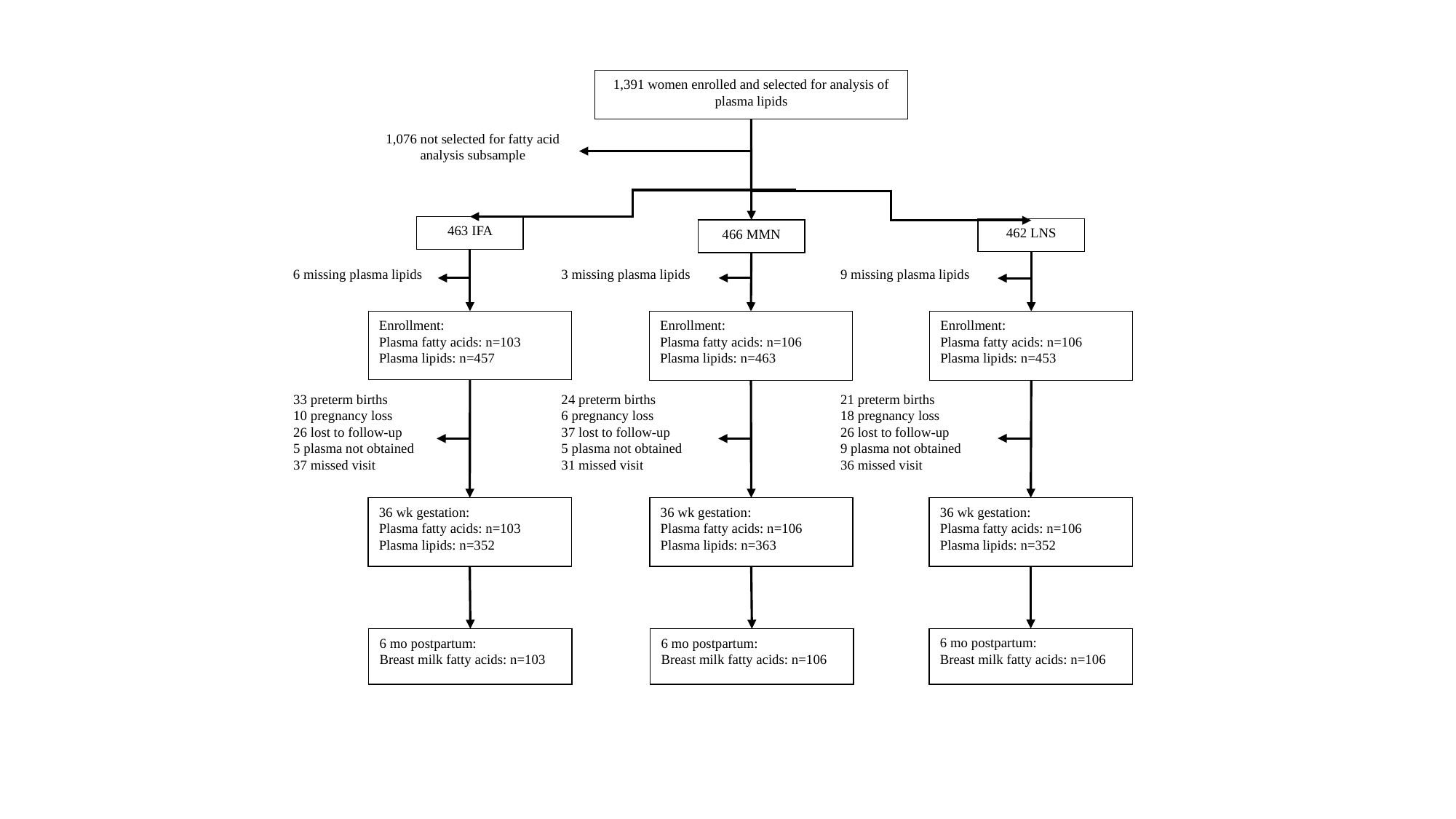

1,391 women enrolled and selected for analysis of plasma lipids
1,076 not selected for fatty acid analysis subsample
462 LNS
466 MMN
6 missing plasma lipids
3 missing plasma lipids
9 missing plasma lipids
Enrollment:
Plasma fatty acids: n=103
Plasma lipids: n=457
Enrollment:
Plasma fatty acids: n=106
Plasma lipids: n=463
Enrollment:
Plasma fatty acids: n=106
Plasma lipids: n=453
33 preterm births
10 pregnancy loss
26 lost to follow-up
5 plasma not obtained
37 missed visit
21 preterm births
18 pregnancy loss
26 lost to follow-up
9 plasma not obtained
36 missed visit
24 preterm births
6 pregnancy loss
37 lost to follow-up
5 plasma not obtained
31 missed visit
36 wk gestation:
Plasma fatty acids: n=103
Plasma lipids: n=352
36 wk gestation:
Plasma fatty acids: n=106
Plasma lipids: n=363
36 wk gestation:
Plasma fatty acids: n=106
Plasma lipids: n=352
6 mo postpartum:
Breast milk fatty acids: n=106
6 mo postpartum:
Breast milk fatty acids: n=103
6 mo postpartum:
Breast milk fatty acids: n=106
463 IFA
