# Supplementary material for: Effects of a lipid-based nutrient supplement during pregnancy and lactation on maternal plasma fatty acid status and lipid profile: Results of two randomized controlled trials
Source: Prostaglandins Leukot Essent Fatty Acids. 2017 Feb;117:28–35. doi: 10.1016/j.plefa.2017.01.007 (PMC5338685; doi:10.1016/j.plefa.2017.01.007)
Supplement: Supplementary file 3 — Supplementary material [file mmc3.docx]

**Supplementary Table 1. Comparison of baseline characteristics of women with missing data vs. women without missing data^a^.**

|  | **Ghana** | | |  | | | **Malawi** | | |
| --- | --- | --- | --- | --- | --- | --- | --- | --- | --- |
| **Characteristic** | **Non-missing data (n=221)** | **Missing data**  **(n=100)** | **P^c^** | |  | **Non-missing data (n=1,067)** | | **Missing data**  **(n=312)** | **P^c^** |
| Maternal age, years | 26.6 | 27.2 | 0.41 | |  | 25.3 | | 23.8 | <0.001 |
| Education, completed years | 7.5 | 6.7 | 0.06 | |  | 4.0 | | 4.1 | 0.90 |
| Primiparous women | 36.2% | 29.0% | 0.21 | |  | 19.7% | | 29.8% | <0.001 |
| Gestational age at enrollment, weeks | 16.1 | 16.5 | 0.25 | |  | 16.8 | | 17.0 | 0.08 |
| Body mass index (BMI), kg/m² | 24.9 | 24.4 | 0.39 | |  | 22.1 | | 22.4 | 0.16 |
| Women with anemia (Hb < 100 g/l) | 11.8% | 16.0% | 0.30 | |  | 18.6% | | 28.5% | <0.001 |
| Women with a positive HIV test^b^ | -- | -- | -- | |  | 12.7% | | 17.5% | 0.04 |
| Women with a positive malaria test (RDT) | 6.8% | 7.0% | 0.94 | |  | 22.8% | | 24.5% | 0.54 |
| ALA, % wt | 0.27 | 0.28 | 0.20 | |  | 0.50 | | 0.52 | 0.89 |
| LA, % wt | 23.2 | 23.2 | 0.94 | |  | 25.3 | | 25.4 | 0.93 |
| DHA, % wt | 5.1 | 4.9 | 0.04 | |  | 4.4 | | 3.6 | 0.16 |
| AA, % wt | 6.3 | 6.1 | 0.18 | |  | 8.1 | | 7.6 | 0.66 |
| n6:n3 | 4.5 | 4.6 | 0.60 | |  | 6.3 | | 7.2 | 0.31 |
| Total cholesterol, mg/dL | 147.9 | 143.8 | 0.45 | |  | 120.5 | | 124.6 | 0.04 |
| HDL-C, mg/dL^b^ | 58.7 | 58.5 | 0.97 | |  | -- | | -- | -- |
| LDL-C, mg/dL^b^ | 63.7 | 61.3 | 0.58 | |  | -- | | -- | -- |
| Triglycerides, mg/dL | 134.8 | 119.5 | 0.07 | |  | 94.8 | | 100.6 | 0.02 |

**^a^** The outcome variables with the largest proportion of missing data were selected for comparison. In Ghana, plasma fatty acids at 36 wk gestation were selected. In Malawi, plasma lipids at 36 wk gestation were selected.

^b^ Women that were HIV positive were not included in the Ghana trial. HDL-C and LDL-C were not measured in the Malawi trial.

^c^ P-values obtained from chi-square test.

**Supplementary Table 2. Median plasma and breast milk fatty acid ratios LA:AA, ALA:DHA, and AA:EPA, by intervention group.**

|  | **Ghana** | | | |  | **Malawi** | | | |  | **Pooled** |
| --- | --- | --- | --- | --- | --- | --- | --- | --- | --- | --- | --- |
|  | **IFA** | **MMN** | **SQ-LNS** | **p^c^** |  | **IFA** | **MMN** | **SQ-LNS** | **p^c^** |  | **p^c^** |
| **LA:AA** |  |  |  |  |  |  |  |  |  |  |  |
| Plasma, Enrollment | 3.8^b^  (3.3, 4.4) | 3.6  (3.2, 4.4) | 3.8  (3.4, 4.4) | 0.33 |  | 3.1  (2.6, 3.5) | 3.4  (2.9, 3.7) | 3.1  (2.7, 3.5) | 0.02 |  | 0.79 |
| Plasma, 36 wk gestation^a^ | 4.5  (3.8, 5.3) | 4.4  (3.8, 5.1) | 4.5  (4.0, 5.3) | 0.56 |  | 3.7  (3.2, 4.4) | 3.9  (3.5, 4.6) | 4.0  (3.4, 4.6) | 0.11 |  | 0.12 |
| Breast milk, 6 mo postpartum | 26.2  (22.7, 30.7) | 26.5  (23.1, 30.7) | 26.7  (22.4, 33.9) | 0.16 |  | 24.8  (20.3, 30.4) | 26.0  (21.8, 32.5) | 26.1  (21.2, 30.7) | 0.20 |  | 0.14 |
| **ALA:DHA** |  |  |  |  |  |  |  |  |  |  |  |
| Plasma, Enrollment | 0.05  (0.04, 0.07) | 0.06  (0.04, 0.07) | 0.05  (0.04, 0.06) | 0.07 |  | 0.11  (0.08, 0.14) | 0.12  (0.09, 0.15) | 0.10  (0.08, 0.14) | 0.19 |  | 0.35 |
| Plasma, 36 wk gestation^a^ | 0.06  (0.05, 0.08) | 0.07  (0.06, 0.09) | 0.07  (0.06, 0.09) | 0.03^d^ |  | 0.13  (0.10, 0.17) | 0.13  (0.10, 0.16) | 0.13  (0.10, 0.16) | 0.20 |  | 0.03^f^ |
|  |  |  |  |  |  |  |  |  |  |  |  |
| Breast milk, 6 mo postpartum | 0.47  (0.33, 0.62) | 0.39  (0.33, 0.54) | 0.48  (0.36, 0.70) | 0.03^e^ |  | 0.91  (0.66, 1.38) | 0.93  (0.64, 1.45) | 1.02  (0.67, 1.70) | 0.56 |  | 0.14 |
| **AA:EPA** |  |  |  |  |  |  |  |  |  |  |  |
| Plasma, Enrollment | 5.2  (3.8, 6.9) | 5.5  (3.8, 7.3) | 4.9  (3.3, 6.8) | 0.38 |  | 13.1  (9.9, 19.6) | 13.7  (10.1, 20.5) | 14.0  (9.2, 20.4) | 0.95 |  | 0.65 |
| Plasma, 36 wk gestation^a^ | 5.3  (3.7, 10.0) | 6.4  (4.7, 10.0) | 5.6  (4.1, 8.3) | 0.37 |  | 17.8  (14.2, 24.1) | 17.0  (12.2, 24.2) | 16.7  (12.3, 22.7) | 0.40 |  | 0.65 |
| Breast milk, 6 mo postpartum | 2.1  (1.3, 3.2) | 2.0  (1.1, 3.0) | 2.2  (1.2, 3.1) | 0.38 |  | 6.0  (4.5, 7.6) | 5.3  (3.7, 7.3) | 5.6  (3.9, 8.5) | 0.21 |  | 0.38 |

^a^ Model includes baseline value of outcome variable for reported p-value.

**^b^** Median (25^th^ percentile, 75^th^ percentile), all such values.

^c^ P-values obtained by ANOVA (baseline plasma and 6 months postpartum breast milk analyses) and ANCOVA (36 wk plasma analyses).

^e^ Two pairwise comparisons had a p-value <0.10: 1) SQ-LNS vs. IFA, Tukey-adjusted p=0.03; and 2)MMN vs. IFA, Tukey-adjusted p=0.08.

^e^ One pairwise comparison had a p-value <0.10: SQ-LNS vs. MMN, Tukey-adjusted p=0.02.

^f^ One pairwise comparison had a p-value <0.10: SQ-LNS vs. MMN, Tukey-adjusted p=0.02.
